# A robotic test of proprioception within the hemiparetic arm post-stroke

**DOI:** 10.1186/1743-0003-11-77

**Published:** 2014-04-30

**Authors:** Lucia Simo, Lior Botzer, Claude Ghez, Robert A Scheidt

**Affiliations:** 1 Department of Physiology, Northwestern University, Chicago, IL, USA; 2 Department of Physical Medicine and Rehabilitation, Northwestern University Feinberg School of Medicine, Chicago, IL, USA; 3 Sensory Motor Performance Program, Rehabilitation Institute of Chicago, Chicago, IL, USA; 4 Department of Biomedical Engineering, Marquette University, Engineering Hall, 342, P.O. Box 1881, Milwaukee, WI 53201-1881, USA; 5 Department of Neuroscience, Columbia University College of Physicians, New York, NY, USA; 6 Department of Neurology, Medical School of Wisconsin, Milwaukee, WI, USA

**Keywords:** Proprioception, Stroke, Detection, Threshold, Uncertainty, Displacement, Force

## Abstract

**Background:**

Proprioception plays important roles in planning and control of limb posture and movement. The impact of proprioceptive deficits on motor function post-stroke has been difficult to elucidate due to limitations in current tests of arm proprioception. Common clinical tests only provide ordinal assessment of proprioceptive integrity (eg. intact, impaired or absent). We introduce a standardized, quantitative method for evaluating proprioception within the arm on a continuous, ratio scale. We demonstrate the approach, which is based on signal detection theory of sensory psychophysics, in two tasks used to characterize motor function after stroke.

**Methods:**

Hemiparetic stroke survivors and neurologically intact participants attempted to detect displacement- or force-perturbations robotically applied to their arm in a two-interval, two-alternative forced-choice test. A logistic psychometric function parameterized detection of limb perturbations. The shape of this function is determined by two parameters: one corresponds to a signal detection threshold and the other to variability of responses about that threshold. These two parameters define a space in which proprioceptive sensation post-stroke can be compared to that of neurologically-intact people. We used an auditory tone discrimination task to control for potential comprehension, attention and memory deficits.

**Results:**

All but one stroke survivor demonstrated competence in performing two-alternative discrimination in the auditory training test. For the remaining stroke survivors, those with clinically identified proprioceptive deficits in the hemiparetic arm or hand had higher detection thresholds and exhibited greater response variability than individuals without proprioceptive deficits. We then identified a normative parameter space determined by the threshold and response variability data collected from neurologically intact participants. By plotting displacement detection performance within this normative space, stroke survivors with and without intact proprioception could be discriminated on a continuous scale that was sensitive to small performance variations, e.g. practice effects across days.

**Conclusions:**

The proposed method uses robotic perturbations similar to those used in ongoing studies of motor function post-stroke. The approach is sensitive to small changes in the proprioceptive detection of hand motions. We expect this new robotic assessment will empower future studies to characterize how proprioceptive deficits compromise limb posture and movement control in stroke survivors.

## Background

Motor impairments are the most frequent and conspicuous deficits that occur after stroke, and many clinical tests have been developed to obtain standardized measures of limb mobility. By some reports however, more than 50% of survivors exhibit somatosensory deficits that negatively impact quality of life and rehabilitation outcome [1-3]. Somatosensory deficits may involve any of the various proprioceptive sensors that signal the physical state of the limb (muscle spindle receptors, Golgi tendon organs and mechanoreceptors in the skin) [4]. Proprioception includes the sense of position, motion and effort. Proprioception of limb kinematics is particularly important for forming the feedforward motor commands that coordinate the arm’s complex nonlinear dynamics during reaching [5-8] and the feedback commands that stabilize the hand against environmental perturbation [9]. Because experimental evidence suggests that these two aspects of control may be differentially affected post-stroke [10], increased understanding of how proprioceptive deficits compromise control of limb posture and movement may prove useful for developing new physical rehabilitation strategies specifically targeting each aspect of control after stroke and for determining which therapeutic approach might best benefit any given patient.

Clinical assessments of proprioception currently suffer poor reliability [11-13] and lack resolution (i.e. they only provide an ordinal classification of proprioceptive integrity: intact, impaired or absent). Consequently, several groups have designed standardized tests [13-16] and automated procedures [17-19] that quantify somatosensory deficits by assessing a person’s ability to identify the static posture of an unseen limb or to indicate when a passively moved limb has changed position. While automated tests can provide greater resolution than clinical tests of proprioception, currently-proposed robotic tests require subjects to actively match the stationary position [17,18,20] or motion [19] of one limb with the other. Limb matching tests require integration of proprioceptive information across the two limbs. Therefore, proprioceptive deficits in either limb as well as deficits in the central integration of that sensory information can compromise test performance. For determining how deficits in proprioceptive perception contribute to motor control deficits, it seems necessary to assess proprioceptive perception within the moving limb itself rather than across limbs.

Here we describe an automated test of proprioceptive integrity in the upper extremity that does not require the integration of sensory information from both arms. Our approach focuses on kinesthetic proprioception by requiring people to detect small position- or force-perturbations applied to one hand within the context of a two-interval, two-alternative forced choice test. We compared this approach to a standard clinical test of proprioception within a small cohort of volunteers to evaluate the ability of the new technique to discriminate individuals with proprioceptive deficits from those without. We identified two ratiometric outcome variables [21] that, when considered together, discriminate individuals with vs. without proprioceptive deficits at least as well as a common clinical test of proprioception. We repeated the robotic assessment on separate days spaced > 1 week apart in an initial evaluation of test-retest reliability. Repeat classification yielded excellent agreement with initial testing but also revealed a subtle learning effect across days of practice on the task. Thus, the robotic procedure we describe can offer a reliable, sensitive and ratiometric test of proprioceptive function.

## Methods

Sixteen unilateral, hemiparetic stroke survivors (SS) and sixteen neurologically intact control (NIC) subjects gave written, informed consent to participate in this study, which was approved by IRB committees at Marquette and Northwestern Universities. Seven SS had clinically-determined proprioceptive deficits and are referred to as SS-P participants. The remaining SS are referred to as SS + P. All SS-P and two SS + P had tactile deficits as well. All SS were recruited from the pool of outpatients at the Rehabilitation Institute of Chicago and ranged in age from 36 to 69 years. Inclusion criteria for SS included upper-extremity Fugl-Meyer (FM) scores ranging between 15–60 (out of 66) (Table 1). Exclusion criteria included presence of neurological or muscular disorders that interfere with neuromuscular function and current use of agents that may interfere with neuromuscular function. NIC subjects (34 to 66 yrs) were age matched (±5 yr) to the stroke survivors.

**Table 1:** Subject characteristics and classifications

**Subject**	**Experiment**	**Age [yrs]**	**Sex**	**Months post-CVA**	**Affected arm**	**Type**	**Lesion site**	**Prop**	**Touch**	**UE FM**	**MAS**	**Paretic grip [kg]**	**Non-paretic grip [kg]**
SS01	D1, D2	52	F	61	L	HEM	—	**A**: MCP	**A**: F, H, FA	21	1.25	2	25
								**I**: W, E, S	**I**: A				
SS02	D1, FT	53	M	36	R	HEM	**L**: TH	**A**: MCP, W	**A**: F, H, FA	20	1.00	6	55
								**I**: E, S	**I**: A				
SS03	D1, D2, FT	50	F	16	R	HEM	**L**: BG	**A**: MCP, W	**A**: F, H, FA	20	1.75	6	23
								**I**: E,S	**I**: A				
SS04^**+**^	D1	50	M	24	L	ISC	**R**: F, T, IN, IC, BG	**I**: MCP, W	**I**: F	17	0.25	7	—
SS05	D1	53	F	42	R	ISC	—	**I**: MCP	**A**: F, H	25	1.00	7	23
									**I**: FA				
SS06	D1, D2, FT	58	M	30	R	ISC	**L**: P, BG	**I**: MCP	**I**: F, H	30	0.50	12	31
SS07	D1	58	M	18	R	ISC	**L**: F, T, P, IN	**I** :MCP	**I**: F	48	0.00	18	—
SS08	D1, D2	65	F	28	R	ISC	**L**: F, P	intact	**I**: F, H	41	0.00	4	25
SS09^*^	FT	54	F	60	R	HEM	L:	intact	**I**: F,H,FA,A	45	0.25	26	36
SS10	D1	59	M	55	R	HEM	—	intact	intact	45	0.25	9	—
SS11	D1	36	M	26	R	ISC	**L**: BG	intact	intact	43	0.50	35	51
SS12	D1, D2, FT	56	F	252	R	ISC	**L**: MCA	intact	intact	28	1.00	8	20
SS13	D1, D2	54	M	69	R	ISC	**L**: IC	intact	intact	48	0.25	31	44
SS14	D1	64	F	74	R	ISC	**L**: IC	intact	intact	21	0.50	3	—
SS15	D1, D2, FT	69	F	240	R	ISC	**L**: F, IC, BG	intact	intact	23	1.25	4	25
SS16	FT	59	F	48	R	ISC	**L**: IC, BG, TH	intact	intact	22	1.50	5	19
NIC01	D1, D2, FT	41	M									48	51
NIC02	D1	38	F									—	—
NIC03	D1, D2	34	F									22	19
NIC04	D1	42	M									51	58
NIC05	D1	42	M									58	55
NIC06	D1	34	M									43	51
NIC07	D1, D2	34	M									53	52
NIC08	FT	34	M									50	44
NIC09	D1, D2, FT	48	M									41	29
NIC10	D1	55	M									41	32
NIC11	D1, D2	66	F									27	22
NIC12	D1	63	F									19	18
NIC13	FT	60	F									22	18
NIC14	FT	60	M									51	49
NIC15	FT	38	M									55	58
NIC16	FT	56	F									23	20

*Abbreviations*: D1 and D2: displacement tests 1 and 2; FT: force test; M: male; F: female; L: Left; R: Right; HEM: hemorrhagic; ISC: ischemic; CVA: cerebral vascular accident; Prop: proprioception; UE: upper extremity; FM: Fugl-Meyer score; MAS: modified Ashworth score; A: absent; I: impaired; TH: thalamus; BG: basal ganglia; F: frontal cortex; T: temporal cortex; P: parietal cortex; IN: insular cortex; IC: internal capsule; MCA: middle cerebral artery; Touch: F: finger tips; H: hand; FA: forearm; A: upper arm. ^*^This participant had paresthesia and mild pain in the involved limb. ^**+**^This participant correctly identified just 70% of the rising tones in the auditory training test. All other participants performed with 85% or better accuracy.

All SS participated in an initial evaluation session wherein the same physician assessed motor function and impairment with the subject seated in an armless chair. Clinical assessments included: visual field/visual search evaluation [22]; the upper extremity portion of the Fugl-Meyer assessment [23]; the modified Ashworth scale (MAS) assessing abnormal muscle tone at the shoulder and elbow - MAS scores were averaged across the two joints and across testing directions (flexion, extension) to estimate abnormal muscle tone in the upper extremity [24]; hand dynamometer grip strength (Fabrication Enterprises, Irvington, NY); and evaluation of proprioceptive and tactile discrimination with the subject’s eyes closed. Proprioception at the shoulder, elbow, wrist, and metacarcophalangeal articulations was evaluated by moving the tested joint up and down (the “*up or down?*” test [25,26]). When the joint stopped moving, the subject was to indicate joint position. Six repetitions were performed at each joint. If response was brisk and accurate (i.e. they made no errors), proprioception was rated “intact”; if the subject was unable to respond with confidence (i.e. they made 1 error), proprioception was rated “impaired”; if the subject was unable to determine position (2 or more errors), proprioception was rated “absent”. Touch was evaluated by the two-point discrimination test using an aesthesiometer [27]. Subjects indicated whether they felt one or two points of contact at finger tips, hand, forearm, and upper arm (six repetitions each); if response was brisk and accurate, tactile discrimination was rated “intact”; if the subject was unable to respond with confidence, it was rated “impaired”; if the subject was unable to discriminate between one and two points, it was rated “absent”.

### Experimental procedures

**Figure 1:**
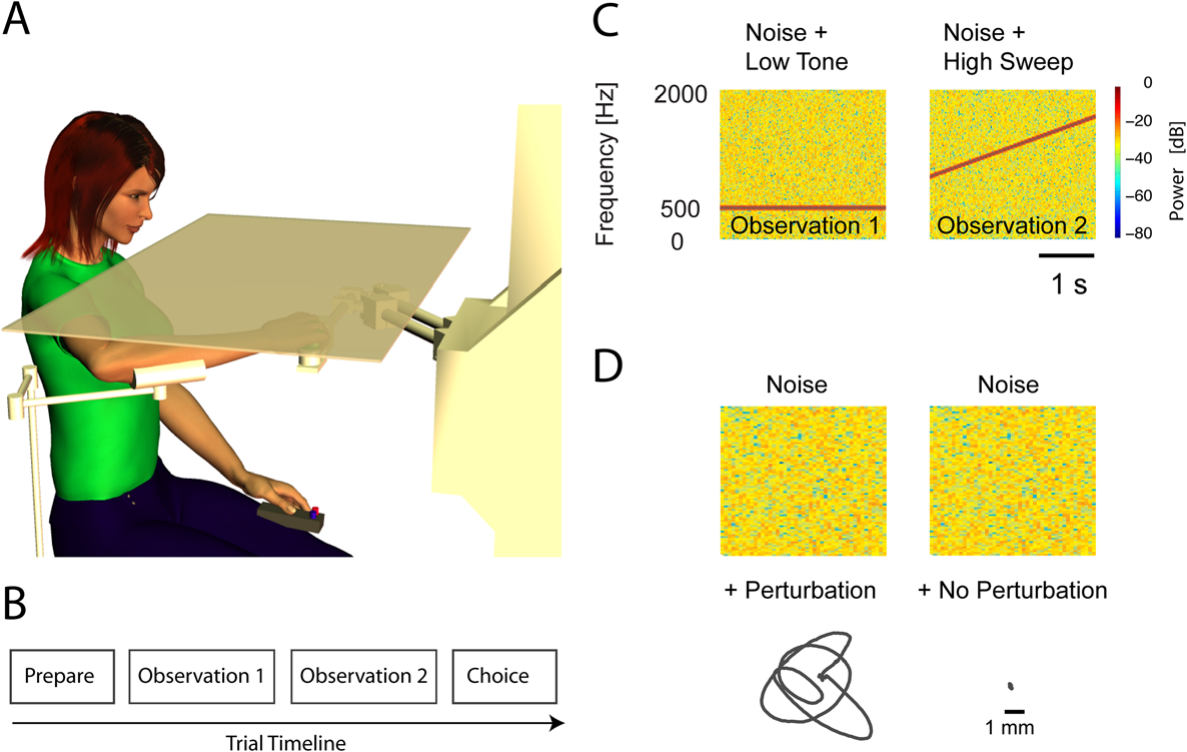
**Experimental setup. A)** Subject seated at the robot. **B)** Trial time-line. **C)** Spectrograms of stimuli used for the training task. **D)** Examples of auditory and mechanical stimuli used in the primary experiment.

Subjects were seated in a high-backed chair. A harness minimized trunk movement. Subjects interacted with a planar robot [28] (Figure 1A). The upper arm was supported against gravity (75° to 90° abduction; ~60° horizontal flexion) using a light-weight, chair-mounted support. Subjects wore a wrist brace on their paretic (SS) or right (NIC) hand to limit motion to the shoulder and elbow. The brace was fixed to the robot handle. An opaque screen placed above the plane of hand motion occluded direct view of shoulder, arm and robot.

#### *Training task*

A tone discrimination task familiarized all participants with the two-alternative forced choice procedure. This task also evaluated each subject’s ability to comprehend multistep instructions, to concentrate, and to use working memory to encode and recall sensory stimuli for comparison in a forced-choice decision process. The task included 24 trials, each of which included two, 3 s observation intervals presented in close succession (Figure 1B). In one interval (randomly selected), a constant-pitch, 500 Hz tone was embedded in auditory white noise (Figure 1C, left). In the other interval, a conspicuously rising pitch was embedded within the noise (Figure 1C, right). Immediately after the second interval, the subject was to indicate via a two-button response box (Figure 1A) whether the first or second noise masked the rising pitch.

#### *Primary experiment: arm movement detection*

14 SS (seven with impaired/absent proprioception) and 11 NIC participated in this experiment. We tested each participant’s ability to detect hand displacements of various magnitudes in a series of 130 trials performed at a single, comfortable location in the center of the arm’s reachable workspace. We programmed the robot to generate whatever force needed (within limits) to enforce desired displacements. Hand displacements were composed of separate sum-of-sinusoids in the “X” (1.75 Hz, 1.2 Hz and 0.25 Hz) and “Y” directions (1.65 Hz, 1.1 Hz and 0.25 Hz). As in the training task, each trial included two observation intervals marked by 3.0 s of auditory white noise and an intervening 1.0 s of silence. One interval (randomly selected) included a perturbation (Figure 1D, left) whereas the other included no perturbation (Figure 1D, right). Participants were instructed to indicate via response box whether the first or second noise masked the hand motion. A fixed set of 9 perturbation magnitudes (*w*) ranged from 0.0 to 1.0 cm (Figure 1D). Each perturbation was presented between 10 and 20 times in pseudorandom order following the Method of Constant Stimuli [29]. To assess test-retest reliability, seven SS and five NIC returned to the lab >1 week later for repeat testing.

#### *Supplemental experiment: hand force detection*

The experimental approach we describe may easily be extended to assess perceptibility of other stimuli commonly used to evaluate motor performance and control following stroke. As a demonstration, we performed a final experiment quantifying detection of force-perturbations applied to the hand. Seven SS (three with impaired or absent proprioception) and seven NIC participants attempted to detect sum-of-sinusoid force perturbations in this single-session experiment comprised of 130 trials. We programmed the robot to generate a specific temporal pattern of desired force vectors regardless of hand position in the workspace. Stimulus detection in this task was therefore driven by controlled hand-force perturbations whereas the magnitude of resulting hand displacement was allowed to vary freely. Nine perturbation magnitudes (*w’s*) spanned the range from 0.0 N to 2.5 N. The within-trial sequence of events and psychophysical task were otherwise as described for the primary experiment. Five of the SS and two of the NIC participants had previously participated in the primary study (see Table 1).

### Data analysis

The training task screened for participants unable to perform the complex psychophysical discrimination task due to an inability to follow multi-step instructions, working memory impairments, and/or attention deficits. We analyzed task performance using an equal-variance Gaussian model of the two-alternative forced-choice task [30]. In that model, the sensations of the signals within the two observation intervals (*X*_*noise*_ , *X*_*signal + noise*_) are drawn from Gaussian distributions with identical variances (σ^*2*^) but different means (*μ*_*noise*_*, μ*_*signal + noise*_). Under the assumption of unbiased observation of the two stimuli, the probability of a positive difference *X*_*signal + noise*_ - *X*_*noise*_ (thus correctly identifying the interval containing signal) is:

(1) $$P_{C}=\Phi \;\left(\frac{\mu_{\text{signal}+\text{noise}}-\mu_{\text{noise}}}{\sqrt{2}\;\sigma^{2}}\right)$$

where *Φ* is the standard cumulative normal distribution function. For each participant, we discarded the first four training trials to account for initial task learning. We then calculated ***P***_***C***_, the percentage of the remaining 20 trials wherein the interval with the rising tone was correctly identified. We defined the limit of acceptable performance as that which would be expected if the sensation variance of the participant under test (i.e. $\sigma_{\text{test}}^{2}$) exceeded that expected of our neurologically-intact participants ($\sigma_{\text{NIC}}^{2}$) by 50% or more. Given the observed variance of NIC performance, Eqn [1] suggests a minimum acceptable performance threshold of 80.4% (n.b. all NIC participants exceeded 90% correct). We therefore considered training task performance ≤80% as indicating potential concerns with the participant’s general ability to perform a two-alternative forced choice test. While other thresholds could have been chosen (for example, allowing acceptable sensation variance to be twice normal yields a performance limit of 73.9%), an 80% threshold seemed a conservative and reasonable limit.

For the motion detection task, we sought to verify the repeatability of robotic hand displacements despite marked variations in arm spasticity across the study population. We measured hand path length during the perturbation then averaged this value across trials, within each perturbation magnitude, for each participant. We also quantified reaction forces induced by the imposed motion using measures of hand force bias and variability. (We defined hand force bias as the average magnitude of horizontal planar hand force during perturbation.) We used these outcome measures to test the hypothesis that spasticity, as assessed by MAS score, would manifest as increased arm stiffness in response to imposed robotic perturbation.

For the primary and supplemental experiments, we characterized proprioceptive sensitivity using *detection threshold* (DT): the minimum magnitude of displacement (or force) that subjects begin to detect reliably when comparing to the no-perturbation condition. We characterized the acuity of proprioceptive sensation using *choice uncertainty* (CU), which quantifies variability of the individual’s responses about the detection threshold.

More specifically, for each participant and each perturbation magnitude on each day, we calculated *P*_*correct*_, the percentage of trials wherein the perturbation interval was correctly identified. Because zero-magnitude perturbations were indistinguishable from their paired comparisons (which were also zero-magnitude), *P*_*correct*_ at zero-magnitude was assigned a likelihood of 0.5. We then fit a cumulative-normal psychometric function (Matlab command: cdf) to *P*_*correct*_ at the 9 perturbation magnitudes using nonlinear optimization (Matlab: fmincon), for each participant on each day:

(2) Pcorrect=0.5+0.5*cdf'normal',DT,CU.

Here, DT was identified as the perturbation magnitude at which the fitted curve passed through 75% likelihood. CU was defined as one standard deviation of the underlying normal distribution. CU values are low when the slope of the psychometric function is steep whereas CU is high when the slope is shallow. The fit was constrained such that: 1) the quantity (DT – CU) > 0.0, and 2) the standard deviation of the underlying normal distribution was greater than 0.002 cm. The first constraint ensures that P(0) is close to 0.5. The purpose of the second constraint is to handle situations where subjects respond with perfect accuracy at and above some perturbation magnitude *w*_*i*_ and at chance for all sample points below *w*_*i*_. In such cases, enforcing a minimum CU coerced the optimization to yield DT values centered within the range bounded by *w*_*i*_ and the next lower magnitude.

### Statistical hypothesis testing

For the primary experiment, we used analysis of variance (ANOVA) and post-hoc Bonferroni t-test to evaluate the effect of proprioceptive integrity (group: NIC, SS + P, SS-P) on performance variables DT and CU obtained during Day 1 testing. We then used repeated-measures ANOVA to test the repeatability of our psychophysical assessment of limb proprioceptive integrity for subjects who returned to the lab for Day 2 testing. For that analysis, we compared parameters DT and CU across testing sessions {Day 1, Day 2} and across the three participant groups (n = 5 NIC; n = 3 SS-P; n = 4 SS + P). We treated “group” and “session” as fixed factors and “subject” as a within-group random factor. For SS, we used linear regression to evaluate correlation between the variables {DT, CU} and performance on the auditory training task. For these participants, we also used multiple linear regression to evaluate correlation between the variables {DT, CU} and the various clinical measures {FM, MAS, Grip Strength}. For the supplemental experiment, we used one-way ANOVA to compare performance measures {DT, CU} across the three groups. Statistical testing was carried out in the Minitab computing environment (Minitab, State College, PA). Effects were considered significant at the α = 0.05 level.

## Results

Table 1 summarizes the clinical test results for the SS group. No participant exhibited visual field deficits or hemispatial neglect. Between the SS-P and SS + P groups, two-sample t-test found no difference in spasticity as quantified by MAS (T = 0.73, p = 0.479). Across groups, all but one participant scored 85% or better on the training task, indicating that they commanded sufficient cognitive resources to perform the forced choice evaluations in the primary and supplemental experiments. We cannot say with confidence whether the remaining subject (SS04) was able to perform two-alternative discrimination tasks reliably because he responded correctly only 70% of the time when presented with contrasting stimuli that were readily discriminated by every other participant. Consequently, we excluded that subject from further statistical analysis.

### Primary experiment: arm movement detection

**Figure 2:**
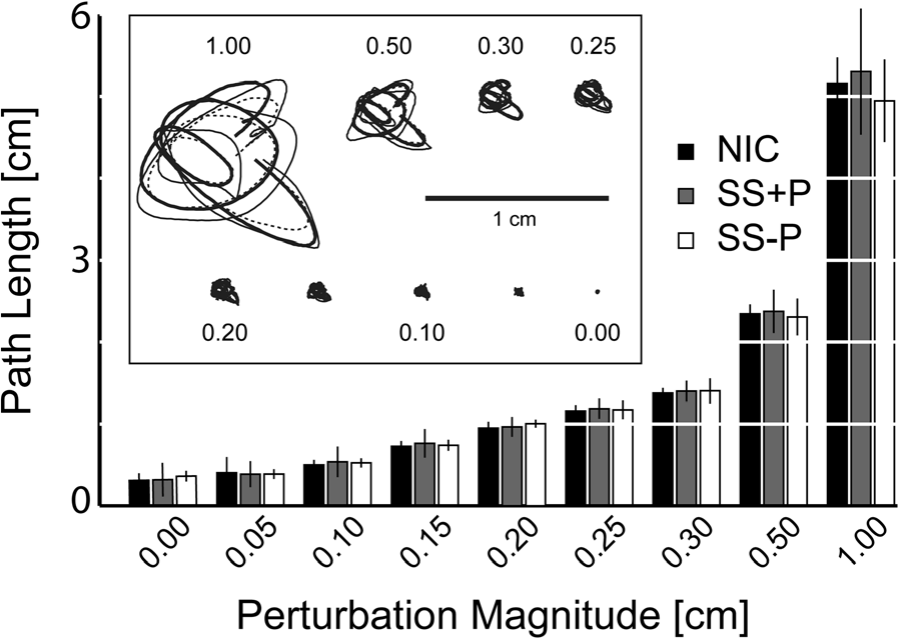
**Hand kinematics in the primary study: Hand paths as a function of perturbation magnitude for each participant group.** Error bars: ±1 SD. Inset: single-trial hand paths randomly selected from among those made by a selected subject from each participant group (NIC: thick solid line; SS + P with MAS of 1.7: thin solid line; SS-P with MAS of 1.0: thin dotted line). The horizontal scale bar within the inset corresponds to 1 cm.

We evaluated the repeatability of robotic perturbation by using repeated measures ANOVA to compare average total hand displacements across the three subject groups and across perturbation magnitudes (Figure 2). Whereas perturbation strength influenced path length [F_(8,224)_ = 1729, p < 0.0005], we found no effect of group [F_(2,224)_ = 0.56, p = 0.57] and no interaction between factors [F_(16,224)_ = 0.87, p = 0.61]. Thus, the robot imposed repeatable displacements (Figure 2, inset) despite large differences in spasticity across groups (Table 1). This finding is likely due to the fact that hand displacements in our test were very small in comparison to the large limb manipulations applied during MAS testing.

A separate repeated measures ANOVA revealed that *mean* hand force varied systematically across groups [F_(2,224)_ = 68.13, p < 0.0005] but not across displacement magnitudes [F_(8,224)_ = 0.03, p = 1.0]. The two SS groups produced more average force against the robot’s handle than did the control group (NIC: 1.33 N ± 0.05 N; SS + P: 2.38 N ± 0.04 N; SS-P: 1.89 N ± 0.05 N). Post-hoc regression analysis found no significant correlation between mean hand force and MAS score (*r*^*2*^ = .01, p = 0.703). Furthermore, repeated measures ANOVA revealed that the within-trial *variability* of hand force (i.e. σ_*f*_) depended systematically on perturbation magnitude as expected [F_(8,224)_ = 70.29, p < 0.0005], but did not vary across subject groups [F_(2,224)_ = 0.18, p = 0.83]. Regression analysis found no correlation between σ_*f*_ and MAS (r^2^ = 0.02, p = 0.510). Because the hand displacement profile was spatially complex, we would have expected σ_*f*_ to increase if the arm’s mechanical stiffness had increased along with the MAS score. These findings suggest that elevated muscle tone in SS manifested as a hypertonic bias at the hand’s testing location rather than a simple increase in the arm’s mechanical stiffness.

**Figure 3:**
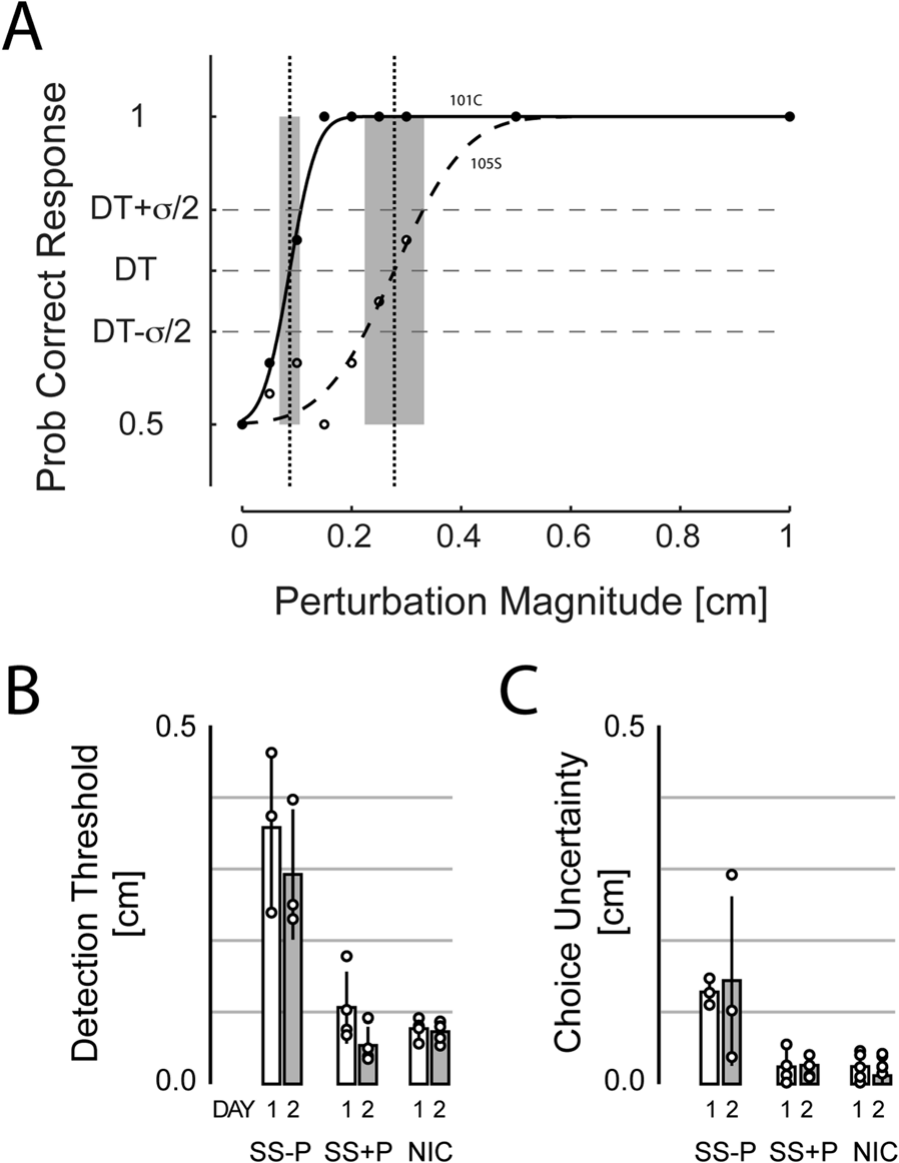
**Primary experimental findings. A)** Cumulative Gaussian psychometric functions fit to observed likelihoods of correct response at each of the applied displacements for selected NIC (filled circles and solid line) and SS-P (open circles and dashed line) participants. Vertical lines indicate movement detection threshold (DT) while shading indicates regions of choice uncertainty (CU); see text for details. **B)** Movement detection threshold (DT) as a function of participant group and testing day for subjects who participated in two days of testing: Day 1: open bars; Day 2: shaded bars. Circles: individual subject data points. **C)** Choice uncertainty (CU) as a function of participant group and testing day. Error bars: ±1 SD.

Participant responses were well-fit by cumulative Gaussian functions of perturbation size (cf. Figure 3A). All participants readily detected large 1.0 cm displacements and all responded with chance accuracy at low perturbation magnitudes. In contrast, responses varied markedly across groups for moderate perturbation magnitudes. For SS, linear regression analyses found no support for a correlation between either of our primary performance measures (DT, CU) and upper extremity FM, MAS or paretic hand-grip scores (p > 0.05 in every case).

A one-way ANOVA of DAY 1 testing revealed a main effect of group on hand motion DT [F_(2,22)_ = 29.31, p < 0.0005]. Post-hoc Bonferroni t-test found the group effect due to higher thresholds in SS-P (0.32 ± 0.11 cm) relative to NIC (0.08 ± 0.03 cm) and SS + P participants (0.09 ± 0.04 cm) (p < 0.0005 in both cases), whose values did not differ from each other (p = 1.000). A separate, planned, one-way ANOVA found a similar main effect of group on choice uncertainty [F_(2, 22)_ = 54.74, p < 0.0005]. Post-hoc Bonferroni t-test revealed that the group effect was due to higher choice uncertainty in the SS-P group (0.14 ± 0.03 cm) relative to the NIC (0.03 ± 0.03 cm) and SS + P (0.02 ± 0.02 cm) groups (p < 0.0005 in both cases), whose values did not differ from each other (p = 1.000).

These outcomes were replicated in separate, two-way, repeated measures ANOVA performed on data from subjects who returned to the lab for a second day of displacement detection testing. Here, we observed a main effect of group on DT [F_(2,9)_ = 29.45, p < 0.0005], a main effect of testing day on DT [F_(1,9)_ = 8.32, p = 0.018] but no interaction between factors [F_(2, 9)_ = 1.89, p = 0.206]. Post-hoc Bonferroni t-test found the group effect due to higher thresholds in SS-P relative to NIC and SS + P participants, whose values did not differ from each other (Figure 3B). We then analyzed test-retest reliability by correlating DT values across days. We found strong correlation (*r* = 0.93) such that *DT*_*day2*_ *= 0 + 0.77 DT*_*day1*_. That is, DT decreased ~0.04 cm from Day1 to Day 2. Because this effect was seen in all three participant groups, it was likely a learning effect mediated by repeat exposure to the task. Note also that this training effect was much smaller than the effect of group, wherein thresholds differed more than 0.2 cm between SS-P and the other two groups.

A separate, planned, two-way repeated measures ANOVA found a main effect of group on choice uncertainty [F_(2, 9)_ = 16.30, p = 0.001] but no effect of testing day [F_(1, 9)_ = 0.05, p = 0.822] or interaction between the factors [F_(2, 9)_ = 0.07, p = 0.933]. After collapsing across days, Bonferroni t-test revealed that the group effect was due to higher choice uncertainty in the SS-P group relative to the NIC and SS + P groups, whose values did not differ from each other (Figure 3C). Test-retest analysis only found a modest correlation of CU values across days (*r* = 0.54) such that *CU*_*day2*_ *= 0.01 + 0.84 CU*_*day1*_.

For subjects who performed above criterion on the auditory training task, linear regression found no evidence of correlation between training task performance and either DT or CU (p > 0.40 in both cases). If we instead include SS04, who performed below criterion, correlation between auditory task performance and CU achieved statistical significance (p = 0.012). Because many factors could lead to impaired performance in two-alternative forced-choice tasks - including failure to follow complex multi-step instructions, memory deficits and attention deficits - inability to perform the auditory test suggests that subject SS04 may have had deficits unrelated to proprioceptive integrity that biased performance on the proprioception test, with choice uncertainty seeming particularly sensitive to confound.

**Figure 4:**
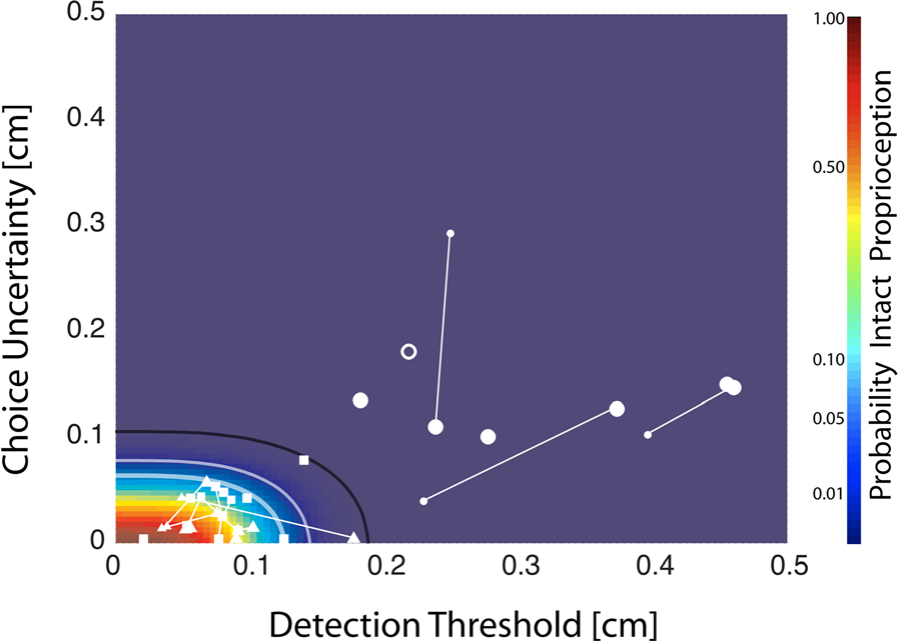
**Plot of DT versus CU for all participants in the primary experimental study (□: NIC; ∆: SS + P; o: SS-P).** For subjects who participated in two days of testing, Day1 results (larger symbols) are connected to Day 2 results (smaller symbols) by a thin line. These data are superimposed on top of a probability map defined as the product of the DT and CU cumulative distribution functions. The curved lines correspond to the 95% (thick white), 99% (thin white) and 99.9% (thin black) confidence bounds on the cumulative, bivariate {DT, CU} distribution of NIC data points.

For NIC subjects, linear regression found no correlation between DT and CT values (r^2^ = 0.07, p = 0.168) and so we estimated the likelihood of intact proprioception at each point in the two-dimensional plane encompassing all {DT, CU} data pairs as the product of the individual DT and CU cumulative likelihood functions from the NIC group (Figure 4, color map). For our cohort of subjects, all NIC and SS + P data points were enclosed within the 99.9% iso-likelihood contour whereas all SS-P data resided outside this same contour. This nonlinear contour yields excellent discriminability between subjects with proprioceptive deficits and those without. In the same way, the normative space established by the bivariate NIC {DT, CU} distribution provides an intuitive interpretation for all points on the {DT, CU} plane in terms of the likelihood of intact proprioception. The displacement detection test was reliable in the sense that retesting > 1 week later did not result in shift from low- to high-likelihood of intact proprioception or *vice-versa*.

### Supplemental experiment: hand force detection

**Figure 5:**
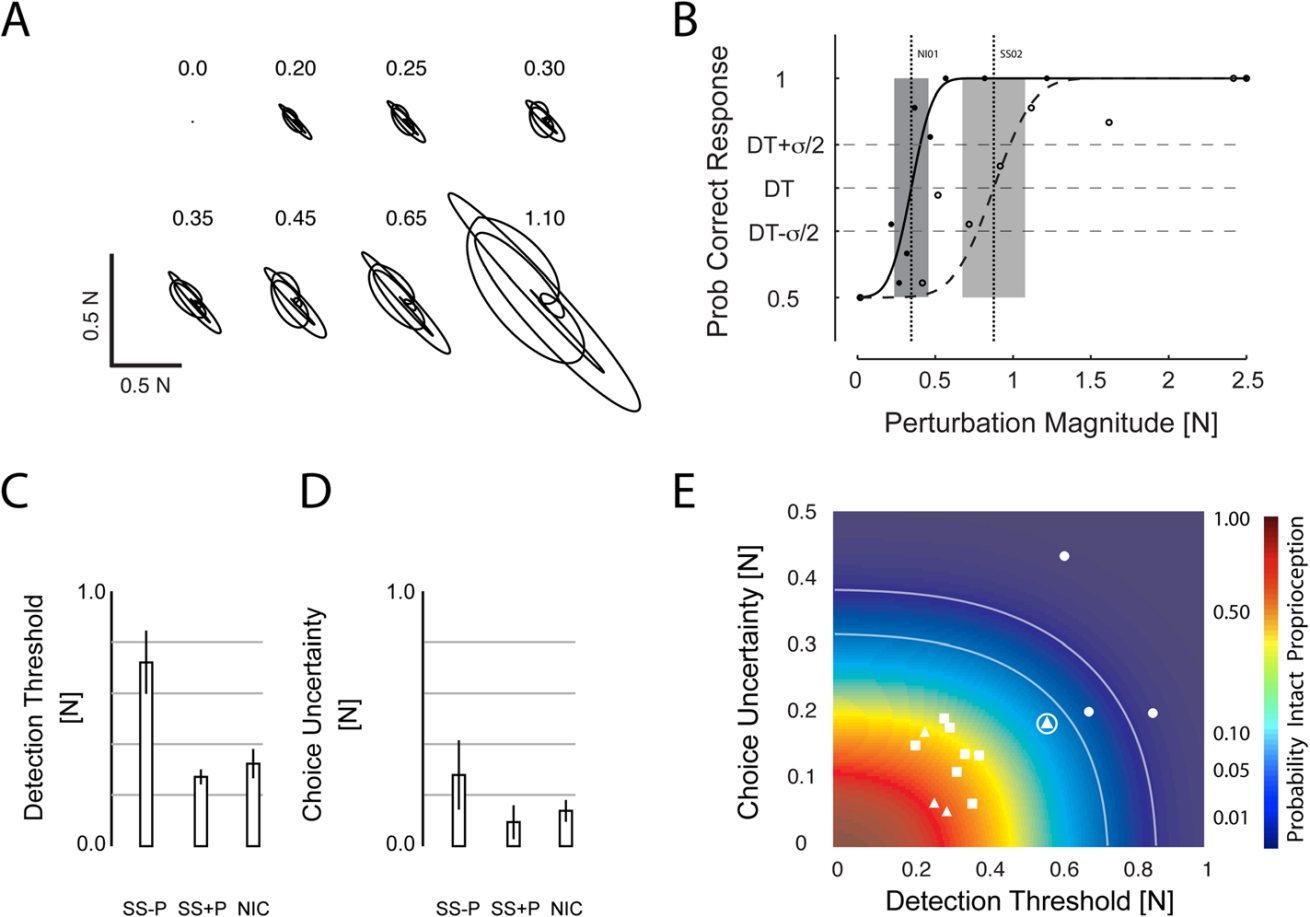
**Supplemental study findings. A)** Spatial profile of imposed hand forces (scale bars: 0.5 N), **B)** Cumulative Gaussian psychometric functions fit to observed likelihoods of correct response at each of the applied force perturbation levels for selected NIC (filled circles and solid line) and SS-P (open circles and dashed line) participants. Other figure elements are as described in Figure 3A. **C)** Hand force detection threshold as a function of participant group. **D)** Choice uncertainty as a function of participant group. In **C)** and **D)**, error bars: ±1 SD. **E)** Plot of DT vs. CU for all participants in the secondary experimental study (□: NIC; ∆: SS + P; o: SS-P). The curved lines correspond to the 95% and 99% confidence bounds on the cumulative bivariate distribution of NIC data points. The encircled triangle corresponds to the results of a SS + P participant with paresthesia and tactile deficits (but no clinical deficits of limb position sense).

We next assessed participant ability to detect a range of hand force perturbations in a two-alternative forced choice experiment (Figure 5A) to determine whether a hand force detection task might also be sensitive to proprioceptive deficits in a small cohort of stroke survivors. Participant responses were again fit by cumulative Gaussian functions of perturbation magnitude (Figure 5B). ANOVA found a main effect of group on hand force DT [F_(2,13)_ = 38.69, p < 0.0005]. Bonferroni t-test revealed that the group effect was due to higher detection thresholds in the SS-P group (0.72 ± 0.12 N) relative to NIC (0.32 ± 0.06 N) and SS + P (0.27 ± 0.03 N) groups (p < 0.0001 in both cases), whose values did not differ from each other (p = 0.957; Figure 5C). A separate ANOVA found a main effect of group on CU [F_(2, 12)_ = 5.20, p = 0.028]. Bonferroni t-test found the group effect due to higher uncertainty in SS-P (0.27 ± 0.14 N) relative to SS + P participants (0.09 ± 0.07 N) (p = 0.039); the difference between the NIC (0.14 ± 0.04 N) and SS-P group did not survive Bonferroni correction (p = 0.065). Choice uncertainty values in the SS + P and NIC groups did not differ from each other (p = 1.00) (Figure 5D). By considering detection threshold and choice uncertainty together, we again found that an iso-likelihood contour could separate SS with proprioceptive deficits from those without (Figure 5E). Although a SS + P participant with tactile deficits, paresthesia, and pain in the arm (SS09; Figure 5E, encircled triangle) had a DT value very close to that of the SS-P participants, this subject’s {DT, CU} data point fell within the 95% confidence bound of the NIC distribution whereas all SS-P data points were outside that contour. Because the bivariate NIC distribution is broader in Figure 5E vs. Figure 4 and because one of the SS-P data points fell within the 99% contour in Figure 5E, empirical results suggest that controlled displacements may provide a better assessment of proprioceptive integrity than force perturbations when using our approach.

## Discussion

Limb movements and environmental interactions are known to be adversely impacted by proprioceptive deficits [10,19,24,31]. However, the causal relationship between proprioceptive deficits and control deficits after stroke is less well understood [32]. Current clinical tests lack resolution to detect small differences in proprioceptive integrity that might reveal, for example, whether there is some sensory threshold below which the likelihood of regaining useful function of the hemiparetic limb is practically small. We therefore developed a psychophysical assessment of proprioceptive integrity using limb displacements and force perturbations, two types of robotic manipulations commonly used to study motor function in stroke survivors. Our approach demonstrates excellent ability to discriminate limbs with impaired or absent proprioception (determined clinically) from those with intact proprioception. The displacement detection test is also reliable; upon retesting > 1 week later, no participant shifted from low- to high-likelihood of intact proprioception or *vice-versa*. Moreover, variations in observed DT and CU values were largely due to participant group membership, although a subtle learning effect was evident across sessions in all participant groups. Thus, the robotic assessment described here can characterize proprioceptive deficits post-stroke with a resolution that is superior to that of common clinical tests. Ultimately, we expect this new approach will be useful for characterizing how impaired proprioception contributes to motor control deficits after stroke.

### Comparison to the “up or down” test of upper extremity proprioception

Current clinical tests of somatosensation post-stroke are useful because they are easy to administer and can give clinicians a quick, rough estimate of a patient’s proprioceptive and tactile integrity. However, the clinical test administered here – the “up or down?” test - is limited in that there are only three possible grades of proprioception: intact, impaired and absent. By collapsing a continuum of impairment onto just three ordinal classes, the clinical test sacrifices measurement resolution for speed and ease of administration. By contrast, the robotic test introduced here yields a pair of ratiometric performance variables (DT, CU) that, when plotted within the normative performance space identified using data from a cohort of NI subjects, indicates the likelihood of proprioceptive impairment in the tested limb as well as a quantitative measure of that impairment.

Another limitation of the “up or down?” test is a ceiling effect [33], whereby subjects who claim impaired proprioceptive perception can nevertheless report the spatial orientation of their elbow and (especially) their shoulder joint accurately and reliably. This is likely due to the unavoidable production of secondary sensory cues by the clinician as she or he moves the proximal limb segments up and down. For example, manipulating the position of a relaxed shoulder can affect the posture of the trunk, cause clothing to shift against the skin, or can cause the head to move slightly. Each of these cues could conceivably be used to infer limb segment orientation. By contrast, manipulations of the wrist and fingers, as well as our robotic test, apply very small perturbations to the hand that are not likely to cause significant motion of the trunk, head or clothing. We measured and reported proprioceptive integrity in the distal limb segments and we grouped all SS with impaired/absent proprioception anywhere in the limb into the SS-P group to avoid this possible confound. The fact that all of the SS-P participants performed poorly on our robotic test – even those with “intact” proprioception at the elbow and/or shoulder - suggests that secondary sensory cues may indeed be a source of confound for the “up or down?” test.

A final advantage of the proposed robotic test over current clinical tests of proprioception is that our test is specifically designed to quantify proprioceptive sensitivity to horizontal planar perturbations similar to those currently used in ongoing studies of robotic interventions for promoting recovery of functional arm movement post-stroke. Future studies seeking to understand the impact of proprioceptive deficits in the control of limb posture and movement post-stroke should quantify proprioceptive sensitivity on a scale commensurate with the environmental perturbations used to challenge sensorimotor performance.

### Comparison to other instrumented tests of upper extremity proprioception

Instrumented tests of arm proprioception fall into two general categories. In the first, one arm is moved passively and unseen to some reachable location and the subject’s task is to actively match the stationary position of the hand [18,20] or configuration of the arm [17] with the other limb. These tests assess a person’s ability to integrate information about the state of both limbs presumably from muscle spindles, which are sensitive to both muscle length and rate of length change. Such tests are ideally suited for assessing the integrity of the entire neuromuscular control arc spanning both limbs. However, limb matching tests are not ideal for determining how proprioceptive deficits in the hemiparetic limb contribute to motor deficits in that same limb. First, some stroke survivors (up to 20%) exhibit proprioceptive deficits in the ipsilesional arm, with most but not all of these also having proprioceptive deficits in their contralesional arm [3]. In addition, some stroke survivors also exhibit subtle motor deficits in their ipsilesional arm [34-36]. As a result, limb matching tests can confound proprioceptive deficits in the arm under evaluation with sensory and motor deficits in the matching limb.

Carey and colleagues have developed a position sense apparatus whereby an examiner moves the unseen wrist to a predefined test position and the subject indicates the perceived wrist angle by aligning a goniometric pointer with an imagined line linking the middle of the wrist and the index finger [14]. Although this approach avoids confounds associated with imitating or matching tasks that involve both limbs, Carey’s test only assesses position sensibility at the wrist. It also lacks automation and does not evaluate the participant’s ability to sense limb motion, which is also important for the control of multijoint movement [37,38].

The second test category requires participants to indicate (verbally, or otherwise) when they detect motion in a slowly moving arm (*threshold to detection of passive motion*) or when the moving limb’s position matches a previously presented position (*passive reproduction of joint position*). Niessen and colleagues used both approaches and found that proprioceptive deficits at the shoulder and abnormalities in scapular kinematics correlate with the presence of shoulder pain [19]. Although the technique used by Niessen appears effective for testing proprioceptive integrity, we believe it possible to improve on their approach by using headphones or acoustic noise to mask audible motor noise from the manipulandum, which can provide subtle cues as to when the device imposes limb motions.

Our experimental design took such considerations into account. Like Carey [14] we avoided confounds associated with matching tasks that involve both limbs by applying perturbations to one limb and using the other only to indicate – via response box – whether the perturbation was present in the first or second observation interval. As in the study by Niessen [19], we used a task in which arm muscles were stretched over a range of magnitudes and velocities rather than a task that assessed joint position sense because muscle spindle receptors respond to dynamic stimuli with large phasic responses that make them particularly well-suited for motion detection [39,40]. Moreover, because subjects were asked to relax their tested arm as the robot applied perturbations, performance in our test was not confounded by motor deficits in either arm. The button box minimized need for verbal communication during testing, which allowed us to use acoustic noise to mask potential robot noises. Finally, we implemented a training task that controlled for deficits in attention, memory or cognition that might confound the assessment of proprioceptive integrity.

### Limitations

Our approach has some limitations. In current clinical practice, therapists use coarse assessments of proprioceptive integrity because they require minimal equipment aside from their own hands and because they are quick to administer. By contrast, the approach described here relies on a costly robot and takes a relatively long time to complete (~45 minutes). Whereas the cost of robotics will decrease over time, testing time must be reduced if the approach is to achieve clinical utility, such as for quantifying subtle week-to-week or month-to-month changes in proprioceptive sensitivity caused by therapeutic intervention. Nevertheless, the proposed robotic assessment will find immediate utility as a research tool because it quantifies proprioceptive sensitivity to limb motion on a ratio scale that is directly commensurate with environmental perturbations used in ongoing studies of rehabilitation robotics. Future studies looking to understand the impact of proprioceptive deficits in the control of limb posture and movement post-stroke should quantify proprioceptive integrity using the same environmental perturbations used to challenge and evaluate motor control and performance. The present study shows how this can be done using both displacement and force perturbations applied to the hand.

Another limitation of our approach derives from our use of a two-alternative forced-choice task to assess proprioceptive integrity. Two-alternative forced-choice tasks require subjects to commit to memory sensory stimuli experienced during an initial observation interval, and to recall those memories for subsequent comparison with stimuli experienced during a second interval. Deficits in comprehension, ability to follow multi-step instructions, attention and/or memory could all negatively impact performance of any two-alternative forced choice task. Here we used an auditory discrimination task to screen participants for cognitive deficits that would impede performance in the proprioception test. We also provided a sound rationale, based on signal detection theory, for excluding individuals from proprioception testing if they exhibit poor performance in the training task. (If hearing loss were suspected, a visual analog of the training task could readily be devised.)

The study itself was limited in the breadth of clinical assessments used to characterize the stroke survivors. We observed a lack of correlation between our two outcome variables (DT, CU) and two common measures of motor impairment (FM and MAS). This is understandable because central lesions that compromise motor pathways can spare sensory pathways and *vice versa*. It would be informative therefore for a future study to determine the extent to which proprioceptive integrity, as measured using the proposed robotic technique, is predictive of other important behavioral characteristics such as arm function [41] and arm use [42] post-stroke. Other future studies could use the proposed test to determine whether the ability of central [43] and peripheral [44] stimulation techniques to enhance motor control derives in part from enhanced central processing of proprioceptive signals or solely by enhancing motor output. Finally, increased understanding of the relationship between proprioceptive deficits and deficits in the control of limb posture [45] and movement [8] may prove useful in identifying patients for whom sensory replacement [46] or augmentation [47] techniques could promote effective control of limb interactions with objects in the environment.

Finally, the current study is limited in that we have tested only a small number of participants. Larger cohorts of participants should be examined to formally evaluate the sensitivity and specificity of the test we introduced here (although it remains unclear which clinical test should be selected as the most appropriate “gold standard” assessment). Additional testing will also refine the normative distributions for DT and CU in NIC individuals, which will facilitate quantification of proprioceptive deficits in stroke survivors both in real (units of [cm]) and relative (% likelihood) terms.

## Conclusions

We described a novel robotic assessment of proprioceptive integrity in the arm using hand displacements and force perturbations, which are commonly used to study motor function in stroke survivors. The new approach demonstrates excellent ability to discriminate limbs with impaired or absent proprioception from those with intact proprioception. The test provides a measure of proprioceptive integrity on a continuous scale that is sensitive to small changes in performance such as learning effects due to repeat performance of the task. Thus, the robotic assessment described here can characterize proprioceptive deficits post-stroke with a resolution superior to that of a common clinical test of proprioception. We expect this new approach will prove useful as a research tool in elucidating how impaired proprioception contributes to motor control deficits after stroke [41].

## Abbreviations

CU: Choice uncertainty; DT: Detection threshold; FM: Fugl-Meyer assessment; IRB: Institutional review board; MAS: Modified Ashworth scale; NIC: Neurologically intact control; RMANOVA: Repeated measures analysis of variance; SD: Standard deviation; SS: Stroke survivor; SS+P: Stroke survivor with intact proprioception; SS-P: Stroke survivor with deficits in proprioception.

## Competing interests

The authors declare no competing interests.

## Authors’ contributions

RAS and CG conceived of the study; LS, LB, CG and RAS participated in data collection; LS and RAS analyzed the data; RAS and LS drafted the manuscript. All authors read and approved the final manuscript.
